# Advances in Recent Nature-Inspired Algorithms for Neural Engineering

**DOI:** 10.1155/2020/7836239

**Published:** 2020-10-28

**Authors:** Ricardo Soto, Juan A. Gómez-Pulido, Eduardo Rodriguez-Tello, Pedro Isasi

**Affiliations:** ^1^Pontificia Universidad Católica de Valparaíso, Valparaíso, Chile; ^2^Universidad de Extremadura, Cáceres, Spain; ^3^Cinvestav Tamaulipas, Soto la Marina, Tamaulipas, Mexico; ^4^Universidad Carlos III de Madrid, Getafe, Spain

Nature-inspired algorithms are general-purpose problem solvers that operate as a collection of intelligent agents, mimicking interesting phenomena from nature in order to efficiently solve a specific problem. Many optimization techniques belonging to artificial intelligence were born under this paradigm, which are able to combine data, knowledge, learning, and search strategies for building advanced algorithms. This is a particularly interesting area for neural engineering and other AI-related applications.

During the last three years, many new nature-inspired algorithms have been proposed, such as human behavior-based optimization, spotted hyena optimization, dragonfly optimization, Andean Condor Algorithm, water evaporation optimization, collective decision optimization, interactive search algorithm, vapour-liquid equilibrium metaheuristic, selfish herds algorithm, scattering and repulsive swarm intelligence, social engineering optimization, virus colony search, thermal exchange optimization, and kidney-inspired algorithm. Most of them involve interesting novel aspects that have enabled the efficient solving of complex problems, particularly from the NP-hard and NP-complete class of problems. In pursuing these aspects and possibilities, there are many trends and open issues that deserve to be investigated.

This special issue presents six original, high-quality articles, clearly focused on theoretical and practical aspects, including cutting-edge topics about neural networks, neural models, brain-computer interface, machine learning, and optimization algorithms.

The first article in this special issue is entitled “Double-Criteria Active Learning for Multiclass Brain-Computer Interfaces” and focuses on improving the data collection process for developing brain-computer interface (BCI) systems. Brain-computer interfaces (BCIs) allow users to control external devices via observed brain activity. Commonly, these systems are constructed by employing electroencephalography (EEG) signal datasets. However, EEG data acquisition is often lengthy and exhaustive because signals often vary during experiments due to both biological and technical causes. In this scenario, a main concern is to develop a robust BCI system but using as few data as possible. In this paper, the authors propose to combine a two-query active learning algorithm with an extreme learning machine (ELM) to solve this problem. The proposed approach is tested on different benchmark datasets, demonstrating that the performance of the proposed hybrid outperforms several state-of-the-art approaches.

The second paper presents a survey about a recent metaheuristic called cat swarm optimization (CSO). The survey focuses on exploring the different variations of CSO as well as on the different application domains where CSO has been successfully used. During the last 10 years, more than 20 interesting variations of CSO have been reported such as binary, multiobjective, parallel, and enhanced parallel CSO. Hybrids involving genetic algorithms, particle swarm optimization, and simulated annealing have also been proposed. Weights, opposition-based learning, and quantum behavior have also been used to complement the basic CSO. In the application context, CSO has mainly been employed in electrical engineering, computer vision, signal processing, and system management. Applications of CSO for wireless devices, petroleum engineering, and civil engineering can also be found in the literature. Finally, CSO is tested on 23 classical benchmark functions and 10 recent benchmark functions confirming the performance of this interesting metaheuristic algorithm.

The third manuscript is called “A Dendritic Neuron Model with Adaptive Synapses Trained by Differential Evolution Algorithm” that proposes to improve the computational efficiency of the dendritic neuron model (DNM) by implementing adaptive synapses (DMAS) which are trained through a differential evolution (DE) algorithm. Extensive experimentation is presented, using five classification datasets from the UCI Machine Learning Repository, for assessing the performance of the proposed DE-DMAS algorithm with respect to those reached by a DNM and a neural network, both trained with the classic backpropagation algorithm. The experimental results in terms of correct rate, convergence rate, ROC curve, and cross-validation confirmed the superiority of DE-DMAS over the other compared algorithms, highlighting certain advantages of applying the self-adaptive synapses proposed in the paper: a stronger robustness, an improved performance of DNM, and a reduction of the input parameters needed by a DNM.

The fourth paper is entitled “A Db-Scan Binarization Algorithm Applied to Matrix Covering Problems” that presents a novel binarization algorithm, based on the db-scan unsupervised machine learning technique, which enables the use of continuous swarm optimization metaheuristics for the efficient solution of discrete combinatorial optimization problems. Using the well-known set covering problem as a case study, the behavior of the proposed db-scan binarization technique is analyzed when it is hybridized with two distinct algorithms: a particle swarm optimization and a cuckoo search. The individual performance of each one of these two hybrid metaheuristics was compared with respect to the one produced by other reference binarization methods employing *k*-means clusters and transfer functions (TFs). The experimental results allow the authors to conclude that the db-scan binarization consistently produced better results, both in terms of solution quality and expended computational time when compared with TFs. With regard to the cluster-based binarization, no significant differences in solution quality could be detected; however, the db-scan binarization significantly improved the convergence times.

The fifth paper is entitled “A Neural Network-Inspired Approach for Improved and True Movie Recommendations” that demonstrates how the application of a recent implementation of neural network improves the intelligent semantic analysis of user reviews and certain external resources in recommender systems. These systems, based on collaborative filtering, allow recommendations to be provided on certain items based on the experience of users when rating them, which finds many interesting applications nowadays, such as movie rating. Moreover, the authors of this article include, in addition to the ratings themselves, multiple external data resources related to user interaction in the context to make an intelligent analysis of the recommendation. The neural network considered is a recurrent implementation that processes the sequence of words semantically with user movie attention, which is a semantic emotion. This way, the recommender system evaluates multivariate movies (ratings, votes, Twitter likes, and reviews) to give a high-accurate recommendation. The system has been satisfactorily tested on a mobile app.

Finally, the article “Image Processing-Based Detection of Pipe Corrosion Using Texture Analysis and Metaheuristic-Optimized Machine Learning Approach” describes an interesting research case where a supervised learning technique adjusted by means of a nature-inspired optimization algorithm is applied to solve an image processing problem. The image processing is used for recognizing corroded and intact pipe surfaces, which is essential to determine current condition of the water supply and waste disposal systems. Nevertheless, the accurate detection is a crucial task where conventional methodologies based on human inspection can be quite inefficient. The intelligent approach constructs a decision boundary for this purpose, applying a support vector machine. In turn, this approach is optimized by using a nature-inspired metaheuristic, the differential flower pollination algorithm, which obtains the best hyperparameters of the support vector machine. As a result, this proposal gives an accuracy rate of 92.8% on a dataset consisting of 2,000 image samples.

